# Carbon accumulation in arid croplands of northwest China: pedogenic carbonate exceeding organic carbon

**DOI:** 10.1038/srep11439

**Published:** 2015-06-19

**Authors:** Xiujun Wang, Jiaping Wang, Minggang Xu, Wenju Zhang, Tinglu Fan, Juan Zhang

**Affiliations:** 1 State Key Laboratory of Desert and Oasis Ecology, Xinjiang Institute of Ecology and Geography, Chinese Academy of Sciences, Urumqi, Xinjiang 830011, China; 2 College of Global Change and Earth System Science, Beijing Normal University, Xinjiekouwai Street No.19, Haidian District, Beijing 100875, and Joint Center for Global Change Studies, Beijing, 100875, China; 3 Earth System Science Interdisciplinary Center, University of Maryland, College Park, MD 20740, USA; 4 College of Agriculture, Shihezi University, Shihezi 832003, Xinjiang, China; 5 Ministry of Agriculture Key Laboratory of Crop Nutrition and Fertilization, Institute of Agricultural Resources and Regional Planning, Chinese Academy of Agricultural Sciences, Beijing 100081, China; 6 Dryland Agriculture Institute, Gansu Academy of Agricultural Sciences, Lanzhou 730070, Gansu, China; 7 School of Resource and Environment, Northeast Agricultural University, Harbin Heilongjiang 150030, China

## Abstract

Soil carbonate (SIC) exceeds organic carbon (SOC) greatly in arid lands, thus may be important for carbon sequestration. However, field data for quantifying carbonate accumulation have been lacking. This study aims to improve our understanding of SIC dynamics and its role in carbon sequestration. We analyzed two datasets of SOC and SIC and their ^13^C compositions , one with over 100 soil samples collected recently from various land uses in the Yanqi Basin, Xinjiang, and the other with 18 archived soil samples from a long-term experiment (LTE) in Pingliang, Gansu. The data from the Yanqi Basin showed that SOC had a significant relationship with SIC and pedogenic carbonate (PIC); converting shrub land to cropland increased PIC stock by 5.2 kg C m^−2^, which was 3.6 times of that in SOC stock. The data from the LTE showed greater accumulation of PIC (21–49 g C m^−2^ year^−1^) than SOC (10–39 g C m^−2^ year^−1^) over 0–20 cm. Our study points out that intensive cropping in the arid and semi-arid regions leads to an increase in both SOC and PIC. Increasing SOC through straw organic amendments enhances PIC accumulation in the arid cropland of northwestern China.

There has been evidence that good land management such as organic amendments can increase soil organic carbon (SOC) in cropland^1^,^2^. Interestingly, a recent report showed that long-term straw incorporation and manure application not only led to SOC enhancement, but also resulted in more carbon sequestration in the form of carbonate in the arid cropland of northern China^3^. Similar finding was previously reported for the Saskatchewan soils in Canada^4^. In addition, there is also evidence of land use and climate change impacts on soil inorganic carbon (SIC) stocks. Particularly, land use has significantly affected SIC levels in cultivated soils in China, showing an increase in northwest China^5^. Similarly, Mikhailova and Post^6^ and Su *et al.*^7^ reported significantly higher SIC stocks in continuously cropped field relative to grassland. These results suggest that there may have been enhancements in SIC stock due to various reasons (e.g., fertilization and straw return into soils), and carbonate may be an important form for carbon sequestration in arid and semi-arid regions that cover about 35% of the Earth’s land surface, thus would play a big role in the global carbon cycle.

Soil inorganic carbon, primarily calcium carbonate, is the most common form of carbon in arid and semi-arid regions. The SIC pool consists of two major components: the lithogenic carbonate (LIC) and pedogenic carbonate (PIC). The former originates as detritus from parent materials, mainly limestone, whereas the latter is formed by dissolution and re-precipitation of LIC or through dissolution of carbon dioxide (CO_2_) into HCO_3_^−^, then precipitation with Ca^2+^ and/or Mg^2+^ originating from non-LIC minerals (e.g., silicate weathering, dust and fertilizers).

While there have been indications of PIC having potential for carbon sequestration and climate mitigation^8^,^9^,^10^, there have been limited studies to quantify carbon sequestration as PIC for the vast arid and semi-arid regions, where the SIC stock is 1–9 times higher than the SOC stock^11^,^12^. On the one hand, some studies indicated that PIC accumulation was extremely low (<3 g C m^−2^ y^−1^) in the arid and semi-arid regions of Canada, USA and New Zealand^4^,^11^. On the other hand, limited studies showed significant PIC accumulation (>10 g C m^−2^ y^−1^) in the arid and semi-arid lands of northern China^3^,^13^. Apparently, more studies are needed to evaluate the PIC dynamics under various land managements.

The News Report entitled “Have desert researchers discovered a hidden loop in the carbon cycle” (http://www.sciencemag.org/content/320/5882/1409.full.pdf), regarding the reports of significant CO_2_ uptake (>100 g C m^−2^ yr^−1^) in deserts^14^,^15^, prompted a great effort to better understand the carbon cycle in the arid region of northwest China. As a part of the effort, a survey was conducted in a typical arid area, the Yanqi Basin that is near the northeast edge of the Taklamakan Desert, China. More than 100 soil samples were collected from desert land, shrub land and cropland, and SOC and SIC stocks were compared between land use types^16^.

In this study, we use data collected from the Yanqi Basin to investigate the impacts of land use change (conversion of shrub land to cropland) on PIC dynamics. In addition, we obtained 18 archived surface soil samples from a controlled long-term experiment (LTE) site in a typical semi-arid region, the Loess Plateau, which was established in 1979 with a mono-cropping system dominated by maize-wheat rotations. There were various fertilization treatments, which can be grouped into with and without organic material addition. Contents of SOC and SIC and their ^13^C compositions were measured for all soil samples. The objective of this work was to study the dynamics of both SOC and PIC in these arid and semi-arid regions, and to evaluate impacts of land management on various soil carbon pools.

## Results

### Surface soil properties in the Yanqi Basin

In general, soil pH was high (7.8–9.1), showing no significant difference between the shrub land and cropland (Table 1). Soil electric conductivity (EC) was much greater in the shrub land than in the desert land and cropland. Overall, extractable Ca and Mg in surface soil were high (>11.7 and >0.22 g kg^−1^) except in the desert lands (<8 and <0.08 g kg^−1^). Surface soil showed a range of 3–16 g kg^−1^ for SOC, and 10–30 g kg^−1^for SIC. Contents of extractable Ca and Mg, SOC and SIC followed the same order: cropland > shrub land > desert land. The δ^13^C value in root showed a wider range (from −23.4‰ to −28.3‰) for C3 plants but a smaller range (from −11.4‰ to −12.6‰) for C4 plant (i.e., maize). The δ^13^C_SOC_ values in surface soil (−21.7‰ to −24.8‰) were close to those in C3 plants. Despite modest difference in the δ^13^C value of root between the shrub land (−25.38‰) and cropland (−20.55‰), the δ^13^C_SOC_ value was similar (close to −24‰) between the two groups. Our estimated abundance of C4 plant varied much widely in the shrub land (from 0 to 29%) than in the cropland (from 2% to 20%). On average, relative contribution of C4 plant to SOC was 12% in the cropland and 10% in the shrub land.

### Soil carbon variations in the Yanqi Basin

**Figure 1:**
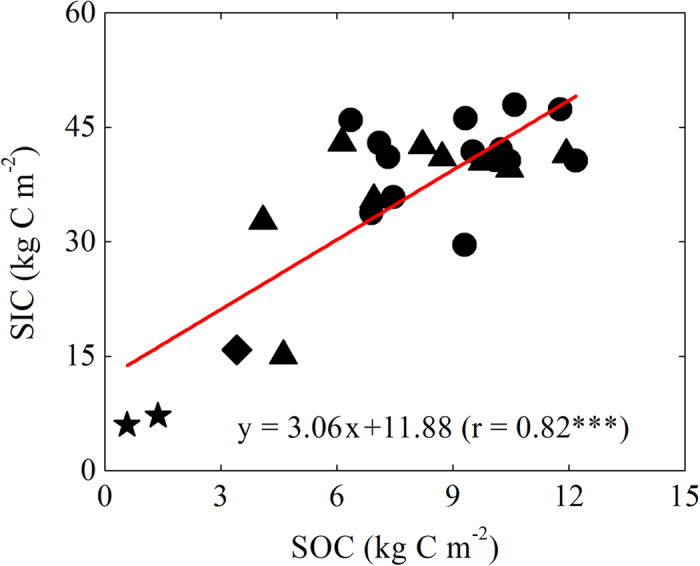
Relationship between soil organic carbon (SOC) and inorganic carbon (SIC) stocks (0–100 cm) for the Yanqi Basin: 2 profiles from desert land (stars), 1 from semi-desert land (diamonds), 9 from shrub land (triangles) and 13 from cropland (circles). Significance of the linear regression was marked with three (P < 0.001) asterisks.

Our data showed large ranges in both SOC (1–12 kg C m^−2^) and SIC (6–45 kg C m^−2^) stocks over the 0–100 cm (Fig. 1). Both the SOC and SIC stocks were significantly higher in the cropland than in the desert land. Although there was no significant relationship between the SOC and SIC stocks in soils collected from the cropland, the whole dataset showed a strong positive correlation (r = 0.80, P < 0.001) between the SOC stock and SIC stock.

**Figure 2:**
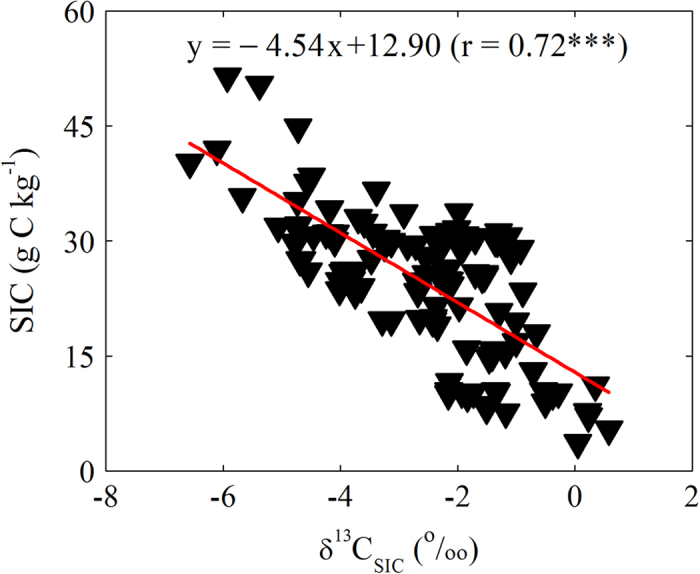
Relationship between SIC content and stable C isotopic composition in SIC (δ^13^C_SIC_). Significance of the linear regression was marked with three (P < 0.001) asterisks.

Figure 2 presented the relationship between SIC content and δ^13^C_SIC_ value using all soil samples, including subsoils. There was a relatively wider range in δ^13^C_SIC_ value, from −6.7‰ to 0.58‰, with the lowest in the cropland and highest in the desert land. Our data exhibited a profound linear relationship (r = 0.72, P < 0.001) between the δ^13^C_SIC_ value and SIC content. Higher SIC contents (>30 g C kg^−1^) were found in those with significant ^13^C depletion (i.e., δ^13^C_SIC_ < −4‰).

**Figure 3:**
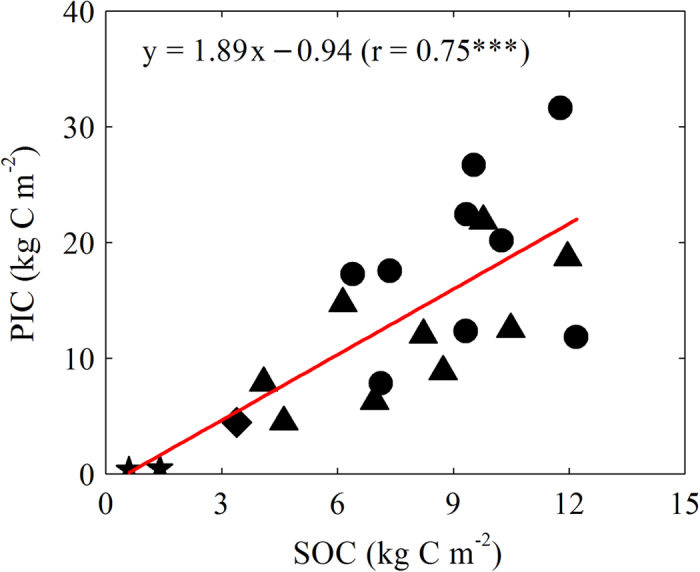
Relationship between pedogenic carbonate (PIC) and SOC stocks (0–100 cm). Significance of the linear regression was marked with three (P < 0.001) asterisks. Symbols are the same as in Figure 1.

We estimated that PIC stock ranged from 0 to 34 kg C m^−2^ over the 0–100 cm. Our data showed a strong correlation (r = 0.75, P < 0.001) between PIC stock and SOC stock (Fig. 3). Overall, PIC stock was higher in the cropland than in the shrub land (also see Table 2). Our analyses suggested that on average, an increase of 1 kg C m^−2^ in the SOC stock was associated with an increase of 1.9 kg C m^−2^ in the PIC stock in this region.

Table 2 illustrated that average SOC stock in the top 0–30 cm was significantly higher in the cropland (4.6 kg C m^−2^) than in the shrub land (3.2 kg C m^−2^), but SOC stock in the subsoil (30–100 cm) was the same (4.6 kg C m^−2^). However, SIC stock showed a much bigger difference in the subsoil (3.9 kg C m^−2^) than in the topsoil (1.2 kg C m^−2^) between the cropland and shrub land. Similarly, difference in PIC was bigger in the subsoil than in the topsoil. Statistical analyses indicted a significant increase of SOC in the topsoil (P < 0.01), but SIC and PIC in the subsoil (P < 0.05) in the cropland. On average, the SOC, SIC and PIC stocks over the 0–100 cm increased by 1.4, 5.1 and 5.2 kg C m^−2^, respectively, as a result of land use change.

### Soil carbon contents and isotopic compositions at the LTE site

**Figure 4:**
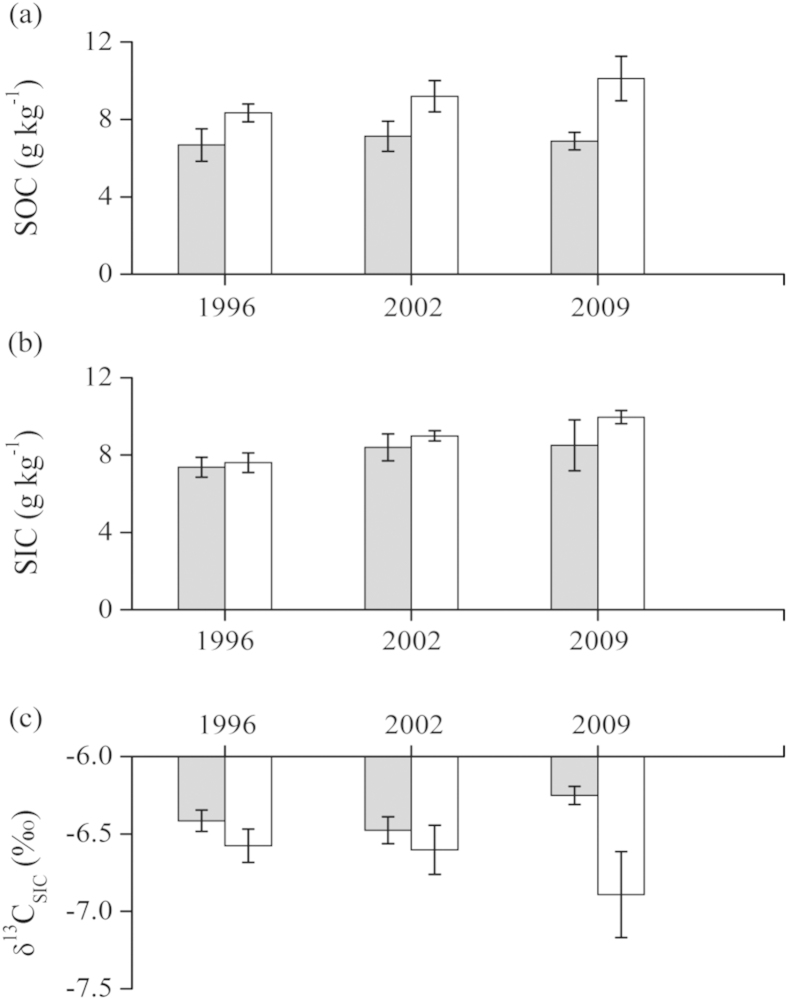
Values and error bars of SOC, SIC and δ^13^C of SIC in soils (0–20 cm) collected in 1996, 2002 and 2009 from the LTE site. (**a**) SOC content, (**b**) SIC and (**c**) δ^13^C of SIC with (empty bars) and without (solid bars) addition of organic materials.

Surface SOC content was significantly higher with addition of organic materials (8.1–10.1 g kg^−1^) than without (6.7–7.1 g kg^−1^) at the LTE site (Fig. 4). Our data revealed an increasing trend in SOC over time in the former but little change in the latter. Surface SIC content was <10 g C kg^−1^ in the Loess, which was much lower than those in the Desert Soil of the Yanqi Basin. There seemed to be no significant difference in surface SIC between the two groups. However, surface SIC showed an increasing trend over time with the addition of organic materials. Overall, there was a positive relationship (r = 0.535, P < 0.05) between the SIC and SOC contents at the LTE site.

The δ^13^C_SIC_ value varied from −6.2‰ to −7.1‰ at the LTE site (Fig. 4), which was close to the most negative values observed in the Yanqi Basin (see Fig. 3). In general, the δ^13^C_SIC_ value was more negative with the addition of organic materials relative to those without. The greatest difference was found in soils collected in 2009. As a result, the difference in δ^13^C_SIC_ value between 2009 and 1996 was much larger with organic material addition than without.

### Carbon stocks and accumulation rates at the LTE site

Surface SOC stock at the LTE was significantly lower than SIC without the addition of organic materials (Table 3). For example, SOC stock in the 0–20 cm remained at ~1.8 kg C m^−2^ during the period of 1996–2009 whereas SIC stock increased from 1.92 kg C m^−2^ in 1996 to 2.21 kg C m^−2^ in 2009. We estimated that PIC stock was in a range of 1.68–1.91 kg C m^−2^. Our analyses suggested that the accumulation rates of SOC, SIC and PIC in the 0–20 cm were 3.5, 21.8 and 9.3 g C m^−2^ y^−1^, respectively.

Long-term application of organic materials resulted in a greater increase in SOC stock relative to SIC stock in the top 20 cm. In particular, SOC stock with addition of organic materials varied from 2.17 kg C m^−2^ in 1996 to 2.63 kg C m^−2^ in 2009, which was significantly higher than those (1.74–1.85 kg C m^−2^) without organic materials addition. While SIC stock was lower than SOC stock, SIC stock showed a greater increasing trend over time, i.e., from 1.98 kg C m^−2^ in 1996 to 2.59 kg C m^−2^ in 2009. Similarly, estimated PIC enhanced from 1.6 kg C m^−2^ in 1996 to 2.15 kg C m^−2^ in 2009. Our analyses showed that long-term application of organic materials led to much greater accumulation rates of SOC, SIC and PIC, i.e., 35.4, 46.6 and 42 g C m^−2^ y^−1^, respectively.

## Discussion

### Relationship between SOC and SIC stocks

Our estimated stocks (0–100 cm) of SOC and SIC are 7.9 and 37 kg C m^−2^ in the shrub land and 9.2 and 42 kg C m^−2^ in the cropland of the Yanqi Basin. The SOC stock (0–20 cm) in the Loess is comparable to that in the shrub land whereas SIC stock is much lower in the former than in the latter. Interestingly, both datasets reveal a strong positive correlation between the SOC stock and SIC stock, which is in disagreement with an earlier report^13^ that showed a negative relationship between SOC and SIC for semi-arid regions in northwest China. However, our finding of positive correlation between the SOC and SIC stocks is consistent with recent reports in soils of China’s arid regions, i.e., near the eastern boundary of Tengger Desert, Inner Mongolia^17^, and at the edge of Badan Jaran Desert, Gansu^7^. These inconsistent findings imply the complex relationship between SOC and SIC because of various processes involved with their accumulations and transformations, and decoupling of these processes over time and space^18^.

The dissolution and precipitation of carbonate involve two main reactions:


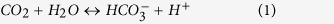



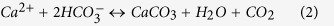


In general, an increase in soil CO_2_ concentration, as a result of SOC decomposition under high SOC level, would lead to production of both 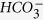 and H^+^. When there is enough H^+^ to create acidic conditions, carbonate dissolution may occur, in which a negative relationship between SOC and SIC is established. On the other hand, the production of 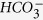 can drive the reaction (2) to the right when there is no Ca limitation, resulting in precipitation of carbonate, which could lead to a positive relationship between SOC and SIC. Soils in arid regions usually have a pH greater than 7.5, and are rich in available Ca^2+^ and/or Mg^2+^. Thus, we postulate that a positive relationship would be more common, particularly in higher pH soils such as in the Yanqi Basin.

### Soil carbonate accumulation rate

There have been a number of studies of the magnitudes and spatial distributions of SIC at regional scales in China^5^,^19^. However, detailed analyses of SIC accumulation are limited. A study of China’s grassland showed that SIC stock in the top 10 cm had declined (at a rate of 26.8 g C m^−2^ year^−1^)^20^ whereas the data from the cropland at the LTE show an increasing trend (32–55 g C m^−2^ year^−1^) for the SIC stock (0–20 cm). There was also evidence of significant accumulation of SIC in other parts of the cropland in northern China^3^. The larger decrease in the SIC stock of the grassland was observed in those locations with stronger soil acidification^20^ whereas the larger increase in the SIC stock of the cropland was found under long-term application of organic materials (see Table 3).

Our estimated PIC accumulation rates in the cropland (>20 g C m^−2^ year^−1^) are significantly higher than previously reported values (<3 g C m^−2^ y^−1^) for Canada, USA and New Zealand^4^,^11^, but comparable to the earlier reported rates (10–40 g C m^−2^ y^−1^) for the Aridisols in the northwest China^13^ and the recent reported rate for the cropland of northern China^3^. The large discrepancy is probably attributed to the differences in various factors between these regions, e.g., Ca^2+^ and Mg^2+^ availability^11^,^21^. Soils in the Mojave Desert and semi-arid region of Canada may be limited by calcium^4^,^21^,^22^ whereas the croplands in northern China may have various sources of Ca and/or Mg, including fertilizers, dust, irrigation water and weathering of calcium/magnesium silicate minerals^3^. The extractable Ca in the Yanqi Basin is significantly higher in the cropland and shrub land than in the desert land (Table 1), indicating that groundwater (accessible in the cropland and shrub land) may play a role in supplying Ca.

### Implications of intensive cropping for soil carbon dynamics

There have been inconsistent finding on the impacts of land use changes for soil carbon dynamics. On the one hand, there is evidence that tillage during farming may cause a decline in SOC in many regions, e.g., temperate and tropical regions^23^,^24^,^25^. On the other hand, recent studies based on some LTEs revealed an increasing trend in SOC of the topsoil in the cropland of arid and semi-arid regions in northwest China^2^,^26^. The decline of SOC in the temperate and tropical regions is due to enhanced decomposition as a result of tillage whereas the increase of SOC in the arid and semi-arid regions is associated with fertilization and irrigation that lead to enhanced plant growth and subsequent increased organic carbon inputs into the topsoil^1^,^27^,^28^.

Our data demonstrate a significant increase of SOC in the topsoil and an increase of PIC in the subsoil following the conversion of shrub land to cropland in the arid region. Given that the cropland in the Yanqi Basin was converted from shrub land 60 years ago, one could estimate the PIC accumulation rate in the cropland by assuming that the difference between the cropland and shrub land was representative of the carbonate accumulation in the cropland over the past 60 years. This approach yielded an accumulation rate of 87 g C m^−2^ year^−1^ for PIC over the 0–100 cm for the cropland in the Yanqi Basin.

There may be some uncertainties in our estimates of PIC stock and accumulation due to the assumptions made the relative distributions of SOC decomposition, root respiration and atmospheric CO_2_ mixing. To assess these uncertainties, we conducted a sensitivity analysis. As demonstrated by Table 4, changing the contribution of SOC decomposition (thus root respiration) to CO_2_ production showed little effect on PIC estimation; increasing the contribution of the atmospheric CO_2_ yielded a higher PIC fraction in the soils of the shrub land (thus a lower accumulation rate of PIC in the cropland). However, the assumption of 30–90% of soil CO_2_ originating from the atmosphere implies that the arid land is a considerable CO_2_ sink, which has been questioned^29^. Nevertheless, despite the uncertainties, our analyses from this study and Wang, *et al.*^30^ indicate that intensive cropping with sound agricultural practice (particularly applications of organic materials) can lead to enhancements of both SOC and carbonate in arid regions. Yet, more studies are still needed to show and quantify carbonate accumulation, and to understand the regulating mechanisms under various environments and time scales.

## Methods

The Yanqi Basin is located on the southeastern flank of the Tianshan Mountain, with a small variation in altitude (from 1030 to 1160 m). Parent material is primarily alluvium, composed of calcareous silt and sand, originating from weathered limestone from the mountain. Main soil types are Brown Desert Soil and Alluvial Soil that are classified as a Haplic Calcisol and Calcaric Cambisol, respectively^31^. Soils are characterized with high pH (from 8 to 9.4) and high sand/silt contents^32^. The sampling area spans both sides of the Kaidu River (Fig. 1), which has various land uses, including desert land, shrub land, and cropland. The shrub land and cropland cover approximately 1000 km^2^ with 75% as cropland that was converted from shrub land 60 years ago, and had been used for farming with regular applications of mineral fertilizers (i.e., urea and calcium superphosphate) and often combined with organic materials (e.g., farmyard manure). The area has an annual precipitation of about 80 mm, annual evaporation of >2000 mm, but abundant groundwater 1–2 m below the land surface. The lands except the desert land have access to groundwater. The croplands have irrigation systems that extract water from the Kaidu River whereas the other lands rely on rainfall and groundwater. Both ground water and river water are rich in various salts, including Ca and Mg ions.

The LTE site has annual precipitation of approximately 540 mm, with 60% from July through September. The soil with a texture of silt loam is classified as Calcarid Regosols^31^. The surface soil had a pH of 8.2, SOC of 6.2 g kg^−1^ and total nitrogen of 0.95 g kg^−1^ prior to the LTE. There are six fertilization treatments: no fertilization, nitrogen fertilization, nitrogen-phosphorus fertilization (NP), manure fertilization, nitrogen fertilization with straw return, and NP fertilization with manure. We group these into two: with (the last three treatments) and without (the first three treatments) organic material addition. Each plot is 16.7 m by 13.3 m with a buffer zone of 1.0 m between plots. Detailed description of the experiment was reported by Fan, *et al.*^26^.

### Soil sampling and analyses

The survey was conducted during August and November, 2010 in the Yanqi Basin, with soil samples and belowground roots collected from various land uses. We randomly selected 25 sites (3 in desert land, 9 in shrub land and 13 in cropland), and sampled one pit (70 cm wide, 100 cm deep) at each site. We collected soil samples from five layers for most soil profiles, i.e., 0–5, 5–15, 15–30, 30–50 and 50–100 cm. Each layer was sampled evenly across the 70 cm width to obtain 1000–2000 g of soil that was then air-dried, thoroughly mixed, and sieved to pass a 2-mm screen. We measured soil physical properties, i.e., water content, bulk density and rock content. Soil pH and EC were measured using a soil:water (1:5) mixture. Exchangeable Ca and Mg were measured by using a flame-atomic absorption spectroscopy with a modified procedure of Mômmik^33^, which included pretreating soils with NH_4_Cl, removing the liquids to eliminate the possible overestimation due to the dissolution of CaCO_3_, and extracting the pretreated soils with 1 M NH_4_Ac. Representative soil and root samples were ground to pass a 0.25 mm screen for analyses of SOC and SIC contents and the stable isotopic compositions in SOC, SIC and root. Detailed procedures were reported by Wang, *et al.*^16^ who presented data from three desert sites, and nine sites each for shrub land and cropland. In this study, we also included SOC and SIC data of four extra profiles from cropland.

The LTE site had archived surface soil (0–20 cm) samples that were collected in fall each year by mixing 6 cores (in 5-cm-diam) of soil in each plot. We obtained samples collected in 1996, 2002 and 2009 with three replicates. SOC and SIC were determined using a CNHS-O analyzer because of small sample size. For SOC measurement, 1 g soil was pretreated with 10 ml 1 M HCl for 12 hours to remove carbonate, then combusted at 1020°C with a constant helium flow carrying pure oxygen to ensure completed oxidation of organic materials. Production of CO_2_ was determined by a thermal conductivity detector. Soil total carbon (STC) was measured using the same procedure without pretreatment of HCl. SIC was calculated as the difference between STC and SOC. Stable carbon isotope was measured using an isotope ratio mass spectrometer (Delta Plus XP, Thermo Finnigan MAT, Germany), with SOC analyzed at the State Key Laboratory of Lake Science and Environment (SKLLSE), Nanjing Institute of Geography and Limnology, Chinese Academy of Sciences (CAS), and SIC at the Nanjing Institute of Geology and Paleontology, CAS^34^. For ^13^C in SOC, CO_2_ was collected in the same way as that for SOC. For ^13^C in SIC, CO_2_ was collected during the reaction of soil with concentrated H_3_PO_4_.

### Estimation of pedogenic carbonate

**Figure 5:**
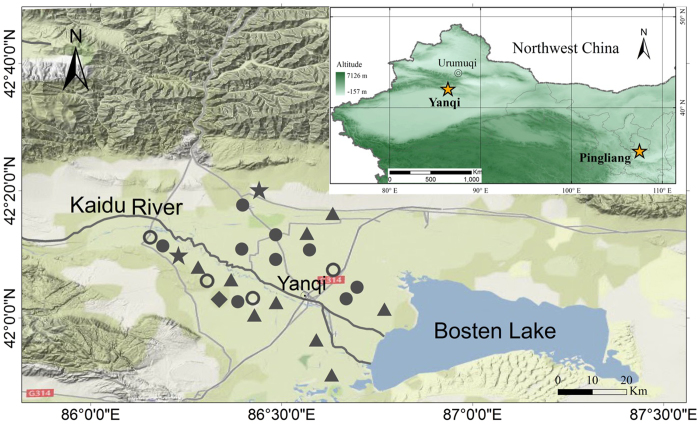
Map and sampling area in the Yanqi Basin and the location of the long-term experiment (LTE) in Pingliang, Gansu. The maps were created using the ARC GIS 9.3. Symbols for the Yanqi Basin are the same as in Figure 1. Soil carbon contents were measured for all profiles, but δ^13^C only determined in those profiles marked with closed symbols.

Following Landi, *et al.*^4^ and Wang, *et al.*^30^ , PIC was calculated as:


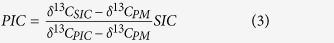


where *δ*^13^*C*_*SIC*_, *δ*^13^*C*_*PM*_ and *δ*^13^*C*_*PIC*_ were the stable ^13^C in carbonate for the bulk SIC, parent material, and pure PIC, respectively. The value of *δ*^13^*C*_*PM*_ was set differently for the two areas. For the Yanqi Basin, we used the largest δ^13^C value (0.58‰) that was obtained from a sample on the desert land to the south of the Kaidu River (see Fig. 5), and similar to these δ^13^C values reported for the surface soils in the Taklamakan Desert^35^. For the LTE site, we set *δ*^13^*C*_*PM*_ as −1‰ that was the highest found in the Loess^36^. Because it was not possible to sample PIC in both areas, we calculated *δ*^13^*C*_*PIC*_ using the following approach.

For the LTE site that is under long-term maize-wheat rotation, *δ*^13^*C*_*PIC*_ was calculated from the stable ^13^C in SOC (*δ*^13^*C*_*SOC*_), according to Wang *et al.*^30^:


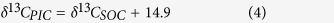


where 14.9 represented the sum of two isotopic fractionation, i.e., diffusion (4.4) and carbonate precipitation (10.5)^37^,^38^,^39^.

For the Yanqi Basin with unknown history of cropping and vegetation, *δ*^13^*C*_*PIC*_ was calculated as:


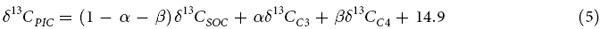


where *δ*^13^*C*_*SOC*_,*δ*^13^*C*_*C3*_ and *δ*^13^*C*_*C4*_ were theδ^13^C in SOC, C_3_ and C_4_ plants, and α and β the relative contributions of C_3_ and C_4_ plants to total CO_2_ production, respectively.

There was evidence that during the growing season, root respiration might exceed SOC decomposition in arid and semi-arid ecosystems^40^,^41^, which was due to low SOC contents. Taking into account that there was no root respiration during non-growing season, it was reasonable to assume that on an annual base, SOC decomposition and root respiration would make equal contribution to CO_2_ production, i.e., α + β = 0.5. Despite uncertainties in this assumption, the δ^13^C_Root_ and δ^13^C_SOC_ values were very close except at three maize sites (see Table 1). Thus, we consider that the assumption would not affect the main results/conclusions.

It is well known that δ^13^C value in SOC provides a means to trace abundance of C_3_/C_4_ vegetation^42^,^43^. Thus, the relative contributions of C_3_ and C_4_ plants were calculated as:


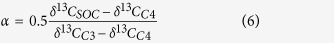



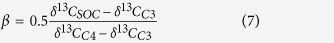


For those sites without data, *δ*^13^*C*_*C3*_ or *δ*^13^*C*_*C*4_ were set as the average values for the Yanqi Basin, i.e., −25.4‰ and −12‰, respectively (see Table 1).

Previous studies indicated that atmospheric CO_2_ might be transferred into soil pores under low rates of soil respiration^37^,^44^,^45^, which would alter the isotopic composition of soil CO_2_. Therefore, for those sites with surface (0–15 cm) SOC less than 7 g kg^−1^ (only six sites: three in the desert land and three in the shrub land), we calculated *δ*^13^*C*_*PIC*_ as:


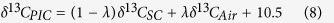



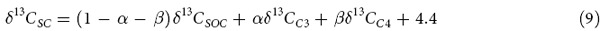


where *δ*^13^*C*_*Air*_ *was* the stable ^13^C composition in the atmospheric CO_2_, and *λ* the proportion of CO_2_ originating from the atmosphere. We set *δ*^13^*C*_*Air*_ to −8‰, and *λ* value to 0.7, 0.5, 0.3 and 0.1 for the 0–5, 5–15, 15–30 and 30–50 cm, respectively.

### Statistical analyses

Linear regression analyses were carried out to evaluate the relationships between various carbon variables (i.e., SOC vs. SIC, SOC vs. PIC, and SIC vs. *δ*^13^*C*_*SIC*_). Student’s t-test was used to determine the significance in the differences in carbon stocks between the cropland and shrub land. All statistical analyses were performed using the SPSS 16.0 software.

## Additional Information

**How to cite this article**: Wang, X.J. *et al.* Carbon accumulation in arid croplands of northwest China: pedogenic carbonate exceeding organic carbon. *Sci. Rep.* **5**, 11439; doi: 10.1038/srep11439 (2015).

## Figures and Tables

**Table 1:** Surface soil pH, electric conductivity (EC), exchangeable Ca and Mg, organic carbon (SOC), inorganic carbon (SIC), and stable^13^C in root and SOC, and estimated abundance of C4 plant for each site.

Vegetation		pH	EC	Ca	Mg	SOC	SIC	δ^13^C_Root_	δ^13^C_SOC_	C4 plant
			(ms cm^−1^)	(g kg^−1^)	(g kg^−1^)	(g kg^−1^)	(g kg^−1^)	(‰)	(‰)	(%)
Desert land	Acroptilon repens (L.) DC.	8.30	0.19	3.92	0.04	3.7	10.3	n.d.	^**−**^21.32	n.d.
	Artemisia desertorum Spreng.	8.41	0.22	7.71	0.07	3.4	16.7	^**−**^23.36	^**−**^21.71	15
Shrub land	Sophora alopecuroides Linn.	8.30	0.97	14.2	0.78	5.7	25.4	^**−**^25.23	^**−**^24.76	4
	Alhagi sparsifolia Shap.	8.72	0.34	11.7	0.83	4.9	24.6	^**−**^25.67	^**−**^23.99	12
	Achnatherum splendens (Trin.) Nevski	8.42	15.0	12.5	0.98	16.8	22.5	^**−**^24.97	^**−**^25.28	0
	Alhagi sparsifolia Shap.	8.44	20.1	13.4	0.73	14.9	23.2	^**−**^23.67	^**−**^25.09	0
	Halostachys caspica C. A. Mey. ex Schrenk	8.26	21.0	13.7	0.66	14.3	17.3	^**−**^23.15	^**−**^24.51	0
	Phragmites australis (Cav.) Trin. ex Steud	7.94	3.35	16.6	0.33	9.3	27.8	^**−**^25.05	^**−**^24.40	5
	Achnatherum splendens (Trin.) Nevski	9.07	6.07	15.3	0.29	5.7	30.1	^**−**^25.60	^**−**^24.09	11
	Halostachys caspica C. A. Mey. ex Schrenk	8.23	8.29	13.3	0.59	7.5	22.3	^**−**^28.29	^**−**^23.52	29
	Tamarix chinensis lour.	8.31	7.06	9.87	0.22	3.4	10.0	^**−**^26.82	^**−**^22.72	28
	Mean	8.41	9.13	13.40	0.60	9.17	22.6	^**−**^25.38	^**−**^24.26	10
	Standard deviation	0.32	7.80	1.96	0.27	4.95	5.9	1.54	0.80	11
Cropland	Capsicum annuum Linn.	8.21	0.18	13.2	0.23	9.5	25.4	^**−**^26.84	^**−**^24.06	19
	Zea mays L.	8.43	0.31	13.5	2.40	14.0	31.3	^**−**^12.57	^**−**^25.17	2
	Gossypium hirsutum Linn.	8.18	0.24	12.7	1.46	14.9	27.7	^**−**^24.70	^**−**^24.41	2
	Beta vulgaris Linn.	8.23	1.11	13.6	0.71	10.7	27.3	n.d.	^**−**^24.03	n.d.
	Capsicum annuum Linn.	7.91	1.36	14.5	1.08	9.5	32.6	^**−**^24.04	^**−**^23.65	3
	Zea mays L.	8.22	0.23	15.8	0.29	11.6	30.5	^**−**^11.90	^**−**^23.47	11
	Helianthus annuus Linn.	8.44	1.57	15.2	0.81	11.7	26.1	^**−**^26.88	^**−**^24.42	17
	Zea mays L.	7.84	1.74	13.2	0.62	11.6	19.7	^**−**^11.41	^**−**^24.23	8
	Brassica campestris L.	8.35	0.53	14.7	1.46	12.6	24.9	^**−**^26.09	^**−**^23.23	20
	Mean	8.20	0.81	14.04	1.01	11.79	27.3	^**−**^20.55	^**−**^24.07	12
	Standard deviation	0.21	0.63	1.05	0.68	1.84	3.9	7.19	0.58	8

**Table 2:** Means and standard deviations (in parentheses) of SOC, SIC, PIC and LIC stocks (kg C m^−2^) for cropland (n = 9) and shrub land (n = 9).

Stock	Cropland		Shrub land		Difference^a^	
	0–30	30–100	0–30	30–100	0–30	30–100
SOC	4.6 (0.6)	4.6 (1.8)	3.2 (1.3)	4.6 (1.7)	1.4**	0
SIC	11.0 (1.5)	30.9 (4.3)	9.8 (2.8)	27.0 (6.9)	1.2	3.9*
PIC	4.4 (1.3)	14.2 (6.7)	3.8 (1.6)	9.6 (5.2)	0.6	4.6*
LIC	6.6 (2.2)	16.7 (5.9)	6.0 (2.3)	17.2 (7.0)	0.6	-0.7
Total C	15.6	35.5	13.0	31.6	2.6	3.9

^a^Significance of the difference was determined by *t* test, and marked with one (P < 0.05) and two (P < 0.01) asterisks.

**Table 3:** Means and standard deviations (in parentheses) of SOC, SIC, PIC and LIC stocks (kg C m ^−2^) and their change rates (g C m^−2^ y^ −1^) and regression coefficients (in parentheses) in 0-20 cm at the LTE.

Stock^a^	1996	2002	2009	Change rate^b^
*Without organic material addition*				
SOC	1.74 (0.22) a	1.85 (0.20) a	1.79 (0.12) a	3.5 (0.17)
SIC	1.92 (0.13) a	2.18 (0.18) a	2.21 (0.34) a	21.8 (0.79)
PIC	1.68 (0.22) a	1.91 (0.15) a	1.81 (0.19) a	9.3 (0.28)
LIC	0.24 (0.09) a	0.27 (0.05) a	0.40 (0.15) a	12.5 (0.91)
*With organic material addition*				
SOC	2.17 (0.12) a	2.39 (0.21) ab	2.63 (0.30) b	35.4 (0.99)
SIC	1.98 (0.13) a	2.34 (0.07) b	2.59 (0.09) c	46.6 (0.98)
PIC	1.60 (0.11) a	1.92 (0.02) b	2.15 (0.14) c	42. (0.98)
LIC	0.38 (0.03) a	0.42 (0.09) a	0.44 (0.07) a	4.6 (0.95)

^a^Carbon stocks followed by the same letter in each row are not significantly different.

^b^Change rate is the slope of change trend of carbon stock over time.

**Table 4:** Estimated PIC percentage and stock over 0–100 cm for cropland and shrub land in the Yanqi Basin, using different values for relative contributions (%) of SOC decomposition (A_SOC_, i.e., 1-a-β) and atmospheric CO_2_ (λ).

A_*SOC*_	λ^a^	Cropland		Shrub land		Difference	Accumulation rate
		%	kg C m^−2^	%	kg C m^−2^	kg C m^−2^	g C m^−2^ y^−1^
50	0	42.4	18.6	29.5	11.9	6.7	112
25	0	41.7	18.2	29.2	11.8	6.4	107
**50**^a^	**10−70**	**42.4**	**18.6**	**36.4**	**13.4**	**5.2**	**87**
50	30−90^b^	42.4	18.6	39.7	14.6	4.0	67
25	30−90	41.7	18.2	39.1	14.4	3.8	63

^a^This row represents the standard calculation used in this study.

^b^λvalues are set as 90%, 70%, 50% and 30% for the 0–5, 5–15, 15–30 and 30–50 cm in those profiles with low SOC (<7 g kg^−1^ in 0–15 cm).

## References

[b1] Minasny B. *et al.* Continuous rice cropping has been sequestering carbon in soils in Java and South Korea for the past 30 years. Global Biogeochemical Cycles 26, GB3027, doi: 3010.1029/2012gb004406, doi: 10.1029/2012gb004406 (2012).

[b2] Zhang W. J. *et al.* Soil organic carbon dynamics under long-term fertilizations in arable land of northern China. Biogeosciences 7, 409–425 (2010).

[b3] Wang X. J. *et al.* Fertilization enhancing carbon sequestration as carbonate in arid cropland: assessments of long-term experiments in northern China. Plant & Soil, doi: 10.1007/s11104-11014-12077-x (2014).

[b4] Landi A., Mermut A. R. & Anderson D. W. Origin and rate of pedogenic carbonate accumulation in Saskatchewan soils, Canada. Geoderma 117, 143–156 (2003).

[b5] Wu H. B., Guo Z. T., Gao Q. & Peng C. H. Distribution of soil inorganic carbon storage and its changes due to agricultural land use activity in China. Agriculture Ecosystems & Environment 129, 413–421, doi: 10.1016/j.agee.2008.10.020 (2009).

[b6] Mikhailova E. A. & Post C. J. Effects of land use on soil inorganic carbon stocks in the Russian Chernozem. Journal of Environmental Quality 35, 1384–1388, doi: 10.2134/jeq2005.0151 (2006). 16825458 10.2134/jeq2005.0151

[b7] Su Y. Z., Wang X. F., Yang R. & Lee J. Effects of sandy desertified land rehabilitation on soil carbon sequestration and aggregation in an arid region in China. Journal of Environmental Management 91, 2109–2116, doi: 10.1016/j.jenvman.2009.12.014 (2010). 20630649 10.1016/j.jenvman.2009.12.014

[b8] Lal R. & Kimble J. M. in Global Climate Change and Pedogenic Carbonate (eds Lal R., Kimble J. M., Eswaran H. & Stewart B. A. ) 1–14 (CRC press, 2000).

[b9] Eshel G., Fine P. & Singer M. J. Total soil carbon and water quality: An implication for carbon sequestration. Soil Science Society of America Journal 71, 397–405, doi: 10.2136/sssaj2006.0061 (2007).

[b10] Manning D. A. C. Biological enhancement of soil carbonate precipitation: passive removal of atmospheric CO2. Mineralogical Magazine 72, 639–649, doi: 10.1180/minmag.2008.072.2.639 (2008).

[b11] Scharpenseel H. W., Mtimet A. & Freytag J. in Global Climate Change and Pedogenic Carbonates (eds Lal R., Kimble J. M., Eswaran H. & Stewart B. A. ) 27–42 (CRC Press, 2000).

[b12] Schlesinger W. H. Carbon storage in the caliche of arid soils- A case study from Arizona Soil Science 133, 247–255, doi: 10.1097/00010694-198204000-00008 (1982).

[b13] Pan G. X. & Guo T. in Global Climate Change and Pedogenic Carbonates (eds Lal R., Kimble J. M., Eswaran H. & Stewart B. A. ) 135–148 (CRC Press, 2000).

[b14] Xie J. X., Li Y., Zhai C. X., Li C. H. & Lan Z. D. CO_2_ absorption by alkaline soils and its implication to the global carbon cycle. Environmental Geology 56, 953–961, doi: 10.1007/s00254-008-1197-0 (2009).

[b15] Wohlfahrt G., Fenstermaker L. F. & Arnone Iii J. A. Large annual net ecosystem CO2 uptake of a Mojave Desert ecosystem. Global Change Biology 14, 1475–1487, doi: 10.1111/j.1365-2486.2008.01593.x (2008).

[b16] Wang J. P., Wang X. J., Zhang J. & Zhao C. Y. Soil organic and inorganic carbon and stable carbon isotopes in the Yanqi Basin of northwestern China. European Journal of Soil Science 66, 95–103, doi: 10.1111/ejss.12188 (2015).

[b17] Zhang N. *et al.* Pedogenic carbonate and soil dehydrogenase activity in response to soil organic matter in Artemisia ordosica community. Pedosphere 20, 229–235, doi: 10.1016/s1002-0160(10)60010-0 (2010).

[b18] Zheng J. *et al.* Perspectives on studies on soil carbon stocks and the carbon sequestration potential of China. Chinese Science Bulletin 56, 3748–3758, doi: 10.1007/s11434-011-4693-7 (2011).

[b19] Li Z. P. *et al.* Assessment of soil organic and carbonate carbon storage in China. Geoderma 138, 119–126, doi: 10.1016/j.geoderma.2006.11.007 (2007).

[b20] Yang Y. H. *et al.* Widespread decreases in topsoil inorganic carbon stocks across China’s grasslands during 1980s-2000s. Global Change Biology 18, 3672–3680, doi: 10.1111/gcb.12025 (2012).

[b21] Monger H. C. & Gallegos R. A. in Global Climate Change and Pedogenic Carbonate (eds Lal R., Kimble J. M., Eswaran H. & Stewart B. A. ) 273–289 (CRC press, 2000).

[b22] Hirmas D. R. & Graham R. C. Pedogenesis and soil-geomorphic relationships in an arid mountain range, Mojave Desert, California. Soil Science Society of America Journal 75, 192–206, doi: 10.2136/sssaj2010.0152 (2011).

[b23] Conant R. T., Paustian K. & Elliott E. T. Grassland management and conversion into grassland: Effects on soil carbon. Ecological Applications 11, 343–355 (2001).

[b24] Murty D., Kirschbaum M. U. F., McMurtrie R. E. & McGilvray H. Does conversion of forest to agricultural land change soil carbon and nitrogen? a review of the literature. Global Change Biol. 8, 105–123, doi: 10.1046/j.1354-1013.2001.00459.x (2002).

[b25] Ogle S. M., Breidt F. J. & Paustian K. Agricultural management impacts on soil organic carbon storage under moist and dry climatic conditions of temperate and tropical regions. Biogeochemistry 72, 87–121, doi: 10.1007/s10533-004-0360-2 (2005).

[b26] Fan T. L., Xu M. G., Song S. Y., Zhou G. Y. & Ding L. P. Trends in grain yields and soil organic C in a long-term fertilization experiment in the China Loess Plateau. Journal of Plant Nutrition and Soil Science 171, 448–457, doi: 10.1002/jpln.200625192 (2008).

[b27] Turner N. C., Molyneux N., Yang S., Xiong Y.-C. & Siddique K. H. M. Climate change in south-west Australia and north-west China: challenges and opportunities for crop production. Crop and Pasture Science 62, 445–456, doi: http://dx.doi.org/10.1071/CP10372 (2011).

[b28] Khan S., Hanjra M. A. & Mu J. Water management and crop production for food security in China: A review. Agric Water Manag 96, 349–360, doi: 10.1016/j.agwat.2008.09.022 (2009).

[b29] Schlesinger W. H., Belnap J. & Marion G. On carbon sequestration in desert ecosystems. Global Change Biology 15, 1488–1490, doi: 10.1111/j.1365-2486.2008.01763.x (2009).

[b30] Wang X. J. *et al.* Fertilization enhancing carbon sequestration as carbonate in arid cropland: assessments of long-term experiments in northern China. Plant and Soil 380, 89–100, doi: 10.1007/s11104-014-2077-x (2014).

[b31] FAO-UNESCO. Soil Map of the World: Revised Legend. (Rome, 1988).

[b32] Wang X. J., Wang J. P. & Zhang J. Comparisons of three methods for organic and inorganic carbon in calcarous soils of northwest China. PloS ONE 7, doi: 10.1371/journal.pone.0044334 (2012). 10.1371/journal.pone.0044334 PMC3432125 22952957

[b33] Nômmik H. Ammonium chloride-imidazole extraction procedure for determining titratable acidity, exchangeable base cations, and cation exchange capacity in soils. Soil Science 118, 254–262 (1974).

[b34] Chang F. *et al.* Sedimentation Geochemistry and Environmental Changes during the Late Pleistocene of Paleolake Qarhan in the Qaidam Basin. J China Univ Geosci 19, 1–8, doi: http://dx.doi.org/10.1016/S1002-0705(08)60019-9 (2008).

[b35] Wang Y. Q., Zhang X. Y., Arimoto R., Cao J. J. & Shen Z. X. Characteristics of carbonate content and carbon and oxygen isotopic composition of northern China soil and dust aerosol and its application to tracing dust sources. Atmospheric Environment 39, 2631–2642, doi: http://dx.doi.org/10.1016/j.atmosenv.2005.01.015 (2005).

[b36] Liu W. G., Yang H., Sun Y. B. & Wang X. L. Delta C-13 values of loess total carbonate: A sensitive proxy for Asian summer monsoon in arid northwestern margin of the Chinese loess plateau. Chemical Geology 284, 317–322, doi: 10.1016/j.chemgeo.2011.03.011 (2011).

[b37] Cerling T. E. The stable isotopic composition of modern soil carbonate and its relationship to climate Earth and Planetary Science Letters 71, 229–240, doi: 10.1016/0012-821x(84)90089-x (1984).

[b38] Cerling T. E., Solomon D. K., Quade J. & Bowman J. R. On the isotopic composition of carbon in soil carbon-dioxide. Geochimica Et Cosmochimica Acta 55, 3403–3405, doi: 10.1016/0016-7037(91)90498-t (1991).

[b39] Cerling T. E., Quade J., Wang Y. & Bowman J. R. Carbon isotopes in soils and paleosols as ecology and paleoecology indicators. Nature 341, 138–139, doi: 10.1038/341138a0 (1989).

[b40] Li Z. G., Wang X. J., Zhang R. H., Zhang J. & Tian C. Y. Contrasting diurnal variations in soil organic carbon decomposition and root respiration due to a hysteresis effect with soil temperature in a Gossypium s. (cotton) plantation. Plant and Soil 343, 347–355, doi: 10.1007/s11104-011-0722-1 (2011).

[b41] Breecker D. O., McFadden L. D., Sharp Z. D., Martinez M. & Litvak M. E. Deep autotrophic soil respiration in shrubland and woodland ecosystems in Central New Mexico. Ecosystems 15, 83–96, doi: 10.1007/s10021-011-9495-x (2012).

[b42] Krull E. G. & Bray S. S. Assessment of vegetation change and landscape variability by using stable carbon isotopes of soil organic matter. Aust J Bot 53, 651–661, doi: 10.1071/BT04124 (2005).

[b43] Boutton T. W., Archer S. R., Midwood A. J., Zitzer S. F. & Bol R. δ^13^C values of soil organic carbon and their use in documenting vegetation change in a subtropical savanna ecosystem. Geoderma 82, 5–41, doi: http://dx.doi.org/10.1016/S0016-7061(97)00095-5 (1998).

[b44] Breecker D. O., Sharp Z. D. & McFadden L. D. Seasonal bias in the formation and stable isotopic composition of pedogenic carbonate in modem soils from central New Mexico, USA. Geological Society of America Bulletin 121, 630-640, doi: 10.1130/b26413.1 (2009).

[b45] Stevenson B. A., Kelly E. F., McDonald E. V. & Busacca A. J. The stable carbon isotope composition of soil organic carbon and pedogenic carbonates along a bioclimatic gradient in the Palouse region, Washington State, USA. Geoderma 124, 37–47, doi: 10.1016/j.geoderma.2004.03.006 (2005).

